# Mesenchymal stem cell therapy for acute radiation syndrome

**DOI:** 10.1186/s40779-016-0086-1

**Published:** 2016-05-12

**Authors:** Risaku Fukumoto

**Affiliations:** Educational-Scientific Center, Faculty of Health Sciences, Medical University of Białystok, ul. Szpitalna 37, 15-295 Białystok, Poland

**Keywords:** Acute radiation syndrome, Mesenchymal stem cell, Cell therapy, Hematopoietic syndrome, Gastrointestinal syndrome, Military medicine

## Abstract

Acute radiation syndrome affects military personnel and civilians following the uncontrolled dispersal of radiation, such as that caused by detonation of nuclear devices and inappropriate medical treatments. Therefore, there is a growing need for medical interventions that facilitate the improved recovery of victims and patients. One promising approach may be cell therapy, which, when appropriately implemented, may facilitate recovery from whole body injuries. This editorial highlights the current knowledge regarding the use of mesenchymal stem cells for the treatment of acute radiation syndrome, the benefits and limitations of which are under investigation. Establishing successful therapies for acute radiation syndrome may require using such a therapeutic approach in addition to conventional approaches.

Human exposure to radiation and the resulting acute radiation syndrome (ARS) have often been associated with accidents, but the contemporary world also faces growing concerns regarding terrorism that may involve the dispersal of enriched radioactive substances. These events may result in substantial casualties. Therefore, there is an urgent need for the development of countermeasures.

The current medical management of ARS remains far from satisfactory; the best available supportive care, including intravenous (IV) hydration, antiemetics, analgesics, antibiotics, and blood transfusions, can achieve an LD_50/60_ value only between 5.5 and 6.5 Gray (Gy) [1]. The reported “successful” pharmacological interventions are radio-protectants, which are administered before radiation exposure, such as that encountered in the event of a nuclear attack; with the exception of the aforementioned supportive care approved by the U.S. Food and Drug Administration, no treatment is administered after radiation exposure, [2]. Administration of bioactive substances, which are given IV in most cases, has also been tested, with limited success. Such substances include cytokines manufactured *in vitro* and designed to control hematopoiesis and local inflammatory responses that are pathogenic at high levels. At present, they are very costly and have been shown to be effective for a limited number of conditions [3]. Thus, none of these proposed countermeasures are ready to be implemented during a mass-casualty scenario, nor does their use appear to be realistic. Nevertheless, these prospective treatments may be more effective in the presence of other types of treatment, such as responsive cell therapy.

Mesenchymal stem cells (MSCs) are beneficial for the treatment of medical conditions requiring tissue regeneration. Although the current clinical use of MSCs is limited to trials for diseases other than ARS, their unique biological features, such as migration, homing, multi-potency, and minimal host rejection, enable their wider application in medicine. Identification of their underlying mechanisms may greatly improve the prospects of using MSC-based therapies for unmet medical needs, including the treatment of ARS.

ARS represents a diverse and complicated pathology, which varies according to the radiation dose and the tissues exposed. Therefore, highly adaptable strategies are required for the treatment of ARS. In humans, a total body irradiation (TBI) dose of 1 Gy or more is considered to be sufficient to cause ARS [4]. Various data support that the LD_50/60_ is between 3.5 and 5.5 Gy [4]. Organ-specific sensitivity to radiation has been frequently discussed. Because of their greater numbers of rapidly proliferating cells, the hematopoietic system and gastrointestinal (GI) tract are considered to be the major target organs for the treatment of ARS. Specific pathologies of these organs are often referred to as hematopoietic syndrome and gastrointestinal (GI) syndrome, respectively. Although both organs contain a large repertoire of cells with different sensitivities to radiation, the consensus is that hematopoietic syndrome becomes evident at a TBI dose of 1 Gy or more, whereas GI syndrome becomes evident at a TBI dose of 5.5 Gy or more [4].

Stem cell therapy has been introduced in the field of regenerative medicine as a highly adaptable transplantation method [5]. The reported advantages of using stem cells include the ability to self-renew, with a differentiation potential determined by the surrounding microenvironment [5]; the low probability of transplant rejection [6]; the feasibility of IV infusion and rapid distribution to the sites of injury because of cell homing properties [7]; and the possibility of *ex vivo* concentration, differentiation, and expansion [8]. Stem cells have gained much attention because of their use in various clinical settings and animal disease models, including ARS, which has prompted continuous investigations aimed at their widespread application in regenerative medicine.

Different types of stem cells have been reported. These include neural stem cells [9], hematopoietic stem cells (HSCs) [10], and multi-potent mesenchymal stem cells (MSCs) with limited differentiation potential. They also exhibit different sensitivities to radiation. For instance, HSCs are extremely sensitive to radiation [11], suggesting that their transplantation may be considered for the treatment of hematopoietic syndrome [12]. Selecting the right types of stem cells at specific proportions appears to be important for their successful application [13].

MSCs are mainly isolated from the bone marrow and are more resistant to radiation than HSCs [11]. Following nearly two hundred phase II/III clinical trials, MSC therapy has emerged as one of the most advanced approaches in the treatment of many disorders; more than 1,400 patients enrolled in clinical trials have received the first marketed formulation of MSC Prochymal® (Osiris Therapeutics, Inc., 7015 Albert Einstein Drive, Columbia, MD 21046 USA), with no report of serious adverse events linked to treatment [14]. It has been established that in many disease models, IV-infused MSCs closely mimic patient resident MSCs that, in response to chemotactic homing signals released from the sites of local injuries, leave the bone marrow, enter the circulation, and travel to sites of tissue damage [15]. Importantly, these infused cells generally retain their properties and reduce inflammation [16], modulate the allogeneic immune response [17], and stimulate hematopoiesis [13, 18]. These are important additions to the already appreciated properties of MSCs, which replenish lost cell types, including those found in the nervous system, skin, bone, fat, cartilage, tendon, muscles (cardiac and skeletal), epithelia (lung, gut, and kidney), and other tissues. A recent study reported that in mice with acute radiation injury, MSCs infused *via* IV exerted protective and therapeutic effects through hematopoietic stimulation [13]. Further mechanistic studies and advances are eagerly awaited.

This article of *Military Medical Research* features the report of Eaton and Varney [19], who illustrate the possible use of MSC therapy for the treatment of ARS from the point of view of military medicine. The effect of MSC therapy on hematopoietic and GI syndrome, as well as cutaneous and combined-injury, is under debate as military personnel are likely to suffer wound trauma. In addition, a practical issue related to actual production timelines is the feasibility of using MSC therapy for military medical management in the event of a nuclear disaster, which is highly unpredictable.

The favorable effects of MSCs on the hematopoietic system have been demonstrated both *in vitro* and *in vivo*. MSCs have been shown to secrete an array of cytokines *in vitro*, which may support the growth of HSCs and their progeny in an *ex vivo* environment [20]. An *in vitro* study found that MSCs support the growth of irradiated CD34^+^ cells [21], which necessitates further investigation to determine an efficacious ratio of HSC-MSC co-transplantation for the treatment of radiation-induced hematopoietic syndrome [22, 23]. Irrespective of the medical disorders treated, patient demographics, and treatment regimen, clinical trials employing HSC grafts generally have revealed better HSC graft rates in the presence of MSCs [24–27]. This is particularly encouraging considering the anticipated limited availability of HSCs under mass-casualty scenarios and situations in which transplanted HSCs are likely to be obtained from two immunologically disparate donors [28, 29]. There has been successful clinical of HSC-MSC co-transplantation following radiation or chemotherapy administered for the treatment of both malignant and non-malignant disorders. Such data support the idea that MSC therapy is a safe and effective treatment modality for facilitating hematopoietic recovery and overall survival following medical interventions that involve HSC transplantation [17].

Clinical trials on the use of MSCs for the treatment of GI diseases have been reported. GI syndrome after radiation exposure is associated with the loss of the rapidly dividing cells, including crypt and immune cells, which are important for epithelial integrity. This loss results in a breach of the epithelial barrier and immune dysregulation, increasing the probability of serious bacterial infection. MSCs have shown significant efficacy in improving certain types of inflammatory bowel diseases, including graft vs. host disease (GvHD) and Crohn’s disease [30, 31]. There has also been a report of the systemic allogeneic infusion of MSCs in accidentally over-irradiated prostate cancer patients with radiation-induced colitis, in which patients experienced a reduction in pain, diarrhea, hemorrhage, inflammation, and fistulization [32]. This evidence, together with the reports of enhanced crypt cell regeneration, restitution of the stem cell niche, and increased xylose absorption following MSC infusion in irradiated animals [33], may be of value in establishing optimal MSC therapy regimens for the treatment of radiation-induced GI syndrome.

The benefits of using MSCs for skin-wound healing in the presence and absence of radiation have been discussed. Regardless of the presence of radiation injury, the administration of MSCs shortens the time for skin-wound healing [34, 35]. Other evidence, including the mobilization of MSCs from the bone marrow in response to wounding [36], has revealed the key roles of MSCs in wound healing.

Collectively, the potential application of MSC therapy for ARS treatment has received much attention and warrants further investigation. Critical reviews, such as this editorial, discussing the outstanding issues regarding the feasibility and safety of MSC-based therapies contributes to the successful management of ARS in the military.

## Conclusion

Treatment of ARS calls for innovative approaches. Although their application is not the front-runner in the treatment of ARS, MSC-based therapies are beneficial for the treatment of various clinical conditions because they directly complement the lost tissues while indirectly creating the microenvironment required for tissue regeneration. Further studies on the efficacy, molecular mechanisms, feasibility, and safety of MSC-based therapies in appropriate models are necessary for the successful management of ARS. Evaluation of the applications of MSCs in the treatment of a broad range of medical conditions, including ARS, will promote improvement in both military and civilian medicine.

## Abbreviations

ARS: acute radiation syndrome
G-CSF: granulocyte colony-stimulating factor
GI: gastrointestinal
GM-CSF: granulocyte/macrophage colony-stimulating factor
GvHD: graft vs. host disease
Gy: gray
HSC: Hematopoietic stem cell
IV: intravenous(-ly)
LD_50/60_: lethal dose of radiation equivalent to 50 % lethality over the course of 60 days
MSC: Mesenchymal stem cell
TBI: total body irradiation

## References

[1] Bushberg JT. Radiation Exposure and Contamination. http://www.merckmanuals.com/professional/injuries_poisoning/radiation_exposure_and_contamination/radiation_exposure_and_contamination.html.

[2] Acute radiation syndrome. http://en.wikipedia.org/wiki/Acute_radiation_syndrome#cite_note-8.

[3] Donnelly EH, Nemhauser JB, Smith JM, Kazzi ZN, Farfan EB, Chang AS (2010). Acute radiation syndrome: assessment and management. South Med J.

[4] Waselenko JK, MacVittie TJ, Blakely WF, Pesik N, Wiley AL, Dickerson WE (2004). Medical management of the acute radiation syndrome: recommendations of the Strategic National Stockpile Radiation Working Group. Ann Intern Med.

[5] Services USDoHH. Regenerative Medicine. http://stemcells.nih.gov/info/scireport/pages/2006report.aspx.

[6] Calkoen FG, Brinkman DM, Vervat C, van Ostaijen-Ten Dam MM, Ten Cate R, van Tol MJ (2013). Mesenchymal stromal cells isolated from children with systemic juvenile idiopathic arthritis suppress innate and adaptive immune responses. Cytotherapy.

[7] Askari AT, Unzek S, Popovic ZB, Goldman CK, Forudi F, Kiedrowski M (2003). Effect of stromal-cell-derived factor 1 on stem-cell homing and tissue regeneration in ischaemic cardiomyopathy. Lancet.

[8] Pittenger MF, Mackay AM, Beck SC, Jaiswal RK, Douglas R, Mosca JD (1999). Multilineage potential of adult human mesenchymal stem cells. Science.

[9] Martino G, Pluchino S (2006). The therapeutic potential of neural stem cells. Nat Rev Neurosci.

[10] Seita J, Weissman IL (2010). Hematopoietic stem cell: self-renewal versus differentiation. Wiley Interdiscip Rev Syst Biol Med.

[11] Sokolov M, Neumann R (2013). Lessons learned about human stem cell responses to ionizing radiation exposures: a long road still ahead of us. Int J Molecul Sci.

[12] Services USDoHH. Hematopoietic Stem Cell Transplant. http://www.remm.nlm.gov/stemcelltransplant.htm.

[13] Hu KX, Sun QY, Guo M, Ai HS (2010). The radiation protection and therapy effects of mesenchymal stem cells in mice with acute radiation injury. Br J Radiol.

[14] ClinicalTrials.gov. https://clinicaltrials.gov.

[15] Wang L, Li Y, Chen X, Chen J, Gautam SC, Xu Y (2002). MCP-1, MIP-1, IL-8 and ischemic cerebral tissue enhance human bone marrow stromal cell migration in interface culture. Hematology.

[16] Meirelles Lda S, Fontes AM, Covas DT, Caplan AI (2009). Mechanisms involved in the therapeutic properties of mesenchymal stem cells. Cytokine Growth Factor Rev.

[17] Tolar J, Le Blanc K, Keating A, Blazar BR (2010). Concise review: hitting the right spot with mesenchymal stromal cells. Stem Cells.

[18] Lange C, Brunswig-Spickenheier B, Cappallo-Obermann H, Eggert K, Gehling UM, Rudolph C (2011). Radiation rescue: mesenchymal stromal cells protect from lethal irradiation. PLoS One.

[19] Eaton EB, Varney TR (2015). Mesenchymal stem cell therapy for acute radiation syndrome: innovative medical approaches in military medicine. Mil Med Res.

[20] Majumdar MK, Thiede MA, Mosca JD, Moorman M, Gerson SL (1998). Phenotypic and functional comparison of cultures of marrow-derived mesenchymal stem cells (MSCs) and stromal cells. J Cell Physiol.

[21] Mourcin F, Grenier N, Mayol JF, Lataillade JJ, Sotto JJ, Herodin F (2005). Mesenchymal stem cells support expansion of in vitro irradiated CD34(+) cells in the presence of SCF, FLT3 ligand, TPO and IL3: potential application to autologous cell therapy in accidentally irradiated victims. Rad Res.

[22] Wang JF, Wu YF, Harrintong J, McNiece IK (2004). Ex vivo expansions and transplantations of mouse bone marrow-derived hematopoietic stem/progenitor cells. J Zhejiang Univ (Sci).

[23] Angelopoulou M, Novelli E, Grove JE, Rinder HM, Civin C, Cheng L (2003). Cotransplantation of human mesenchymal stem cells enhances human myelopoiesis and megakaryocytopoiesis in NOD/SCID mice. Exp Hematol.

[24] Fouillard L, Bensidhoum M, Bories D, Bonte H, Lopez M, Moseley AM (2003). Engraftment of allogeneic mesenchymal stem cells in the bone marrow of a patient with severe idiopathic aplastic anemia improves stroma. Leukemia.

[25] Fouillard L, Chapel A, Bories D, Bouchet S, Costa JM, Rouard H (2007). Infusion of allogeneic-related HLA mismatched mesenchymal stem cells for the treatment of incomplete engraftment following autologous haematopoietic stem cell transplantation. Leukemia.

[26] Meuleman N, Tondreau T, Ahmad I, Kwan J, Crokaert F, Delforge A (2009). Infusion of mesenchymal stromal cells can aid hematopoietic recovery following allogeneic hematopoietic stem cell myeloablative transplant: a pilot study. Stem Cells Dev.

[27] Ball LM, Bernardo ME, Roelofs H, Lankester A, Cometa A, Egeler RM (2007). Cotransplantation of ex vivo expanded mesenchymal stem cells accelerates lymphocyte recovery and may reduce the risk of graft failure in haploidentical hematopoietic stem-cell transplantation. Blood.

[28] Maitra B, Szekely E, Gjini K, Laughlin MJ, Dennis J, Haynesworth SE (2004). Human mesenchymal stem cells support unrelated donor hematopoietic stem cells and suppress T-cell activation. Bone Marrow Transpl.

[29] Kim DW, Chung YJ, Kim TG, Kim YL, Oh IH (2004). Cotransplantation of third-party mesenchymal stromal cells can alleviate single-donor predominance and increase engraftment from double cord transplantation. Blood.

[30] Ringden O, Uzunel M, Rasmusson I, Remberger M, Sundberg B, Lonnies H (2006). Mesenchymal stem cells for treatment of therapy-resistant graft-versus-host disease. Transplantation.

[31] Duijvestein M, Vos AC, Roelofs H, Wildenberg ME, Wendrich BB, Verspaget HW (2010). Autologous bone marrow-derived mesenchymal stromal cell treatment for refractory luminal Crohn’s disease: results of a phase I study. Gut.

[32] Voswinkel J, Francois S, Simon JM, Benderitter M, Gorin NC, Mohty M (2013). Use of mesenchymal stem cells (MSC) in chronic inflammatory fistulizing and fibrotic diseases: a comprehensive review. Clin Rev Allergy Immunol.

[33] Saha S, Bhanja P, Kabarriti R, Liu L, Alfieri AA, Guha C (2011). Bone marrow stromal cell transplantation mitigates radiation-induced gastrointestinal syndrome in mice. PLoS One.

[34] Kim JW, Lee JH, Lyoo YS, Jung DI, Park HM (2013). The effects of topical mesenchymal stem cell transplantation in canine experimental cutaneous wounds. Vet Dermatol.

[35] Hao L, Wang J, Zou Z, Yan G, Dong S, Deng J (2009). Transplantation of BMSCs expressing hPDGF-A/hBD2 promotes wound healing in rats with combined radiation-wound injury. Gene Ther.

[36] Mansilla E, Marin GH, Drago H, Sturla F, Salas E, Gardiner C (2006). Bloodstream cells phenotypically identical to human mesenchymal bone marrow stem cells circulate in large amounts under the influence of acute large skin damage: new evidence for their use in regenerative medicine. Transplant Proc.

